# Trajectories of Pain and Function after Primary Hip and Knee Arthroplasty: The ADAPT Cohort Study

**DOI:** 10.1371/journal.pone.0149306

**Published:** 2016-02-12

**Authors:** Erik Lenguerrand, Vikki Wylde, Rachael Gooberman-Hill, Adrian Sayers, Luke Brunton, Andrew D. Beswick, Paul Dieppe, Ashley W. Blom

**Affiliations:** 1 Musculoskeletal Research Unit, School of Clinical Sciences, University of Bristol, Bristol, United Kingdom; 2 Medical School, University of Exeter, Exeter, United Kingdom; Universidad Europea de Madrid, SPAIN

## Abstract

**Background and Purpose:**

Pain and function improve dramatically in the first three months after hip and knee arthroplasty but the trajectory after three months is less well described. It is also unclear how pre-operative pain and function influence short- and long-term recovery. We explored the trajectory of change in function and pain until and beyond 3-months post-operatively and the influence of pre-operative self-reported symptoms.

**Methods:**

The study was a prospective cohort study of 164 patients undergoing primary hip (n = 80) or knee (n = 84) arthroplasty in the United Kingdom. Self-reported measures of pain and function using the Western Ontario and McMaster Universities Osteoarthritis index were collected pre-operatively and at 3 and 12 months post-operatively. Hip and knee arthroplasties were analysed separately, and patients were split into two groups: those with high or low symptoms pre-operatively. Multilevel regression models were used for each outcome (pain and function), and the trajectories of change were charted (0–3 months and 3–12 months).

**Results:**

Hip: Most improvement occurred within the first 3 months following hip surgery and patients with worse pre-operative scores had greater changes. The mean changes observed between 3 and twelve months were statistically insignificant. One year after surgery, patients with worse pre-operative scores had post-operative outcomes similar to those observed among patients with less severe pre-operative symptoms. Knee: Most improvement occurred in the first 3 months following knee surgery with no significant change thereafter. Despite greater mean change during the first three months, patients with worse pre-operative scores had not ‘caught-up’ with those with less severe pre-operative symptoms 12 months after their surgery.

**Conclusion:**

Most symptomatic improvement occurred within the first 3 months after surgery with no significant change between 3–12 months. Further investigations are now required to determine if patients with severe symptoms at the time of their knee arthroplasty have a different pre-surgical history than those with less severe symptoms and if they could benefit from earlier surgical intervention and tailored rehabilitation to achieve better post-operative patient-reported outcomes.

## Introduction

Joint replacement is a common elective surgical procedure, with more than 158,000 primary hip and knee arthroplasties performed during 2013 in England, Wales and Northern Ireland [1]. In 2006, the estimated age-standardised rates of primary hip and knee arthroplasty in the UK were 0.14% for women and 0.10% for men[2]. In 2005 the lifetime risk of hip arthroplasty was 11.6% for women and 7.1% for men, and for knee arthroplasty the lifetime risks were 10.8% for women and 8.1% for men [3]. These probably reflect underestimates as the number of hip and knee arthroplasty have increased from 2005 to 2013 by 90% and 97% respectively [1]. The main aim of these procedures is to relieve pain and disability, and most, but not all, patients report a good outcome after their surgery [4].

Many studies have reported important improvement in patient-reported outcomes by comparing scores completed at baseline and at one single post-operative assessment usually 6 or twelve months after the arthroplasty[5]. Findings on the extent and timing of pain and functional gains made during the first 12 post-operative months show that most of the recovery occurs within the first three months [6–16]. While several studies have found no change or minor changes in pain and function after this early recovery period [6, 9–11, 15, 16] improvements beyond this three months period have also been reported [6–8, 12–14]. Few studies [8, 12–14] used statistical tests to ascertain the existence of any change between 3 and 12 months after surgery.

Known determinants of post-operative outcome following hip and knee arthroplasties include patients’ expectations, mental health status, co-morbidities and the severity of pre-operative pain and function [17–25]. Patients reporting the worst pre-operative scores have been found to report worse scores 6 and 12 months after surgery, but may have greater benefit from their surgery than those with better pre-operative scores [11, 15, 17, 24, 26–32]. Among the few studies which have reported pain and function measured at three to four months post-operatively by level of pre-operative score [11, 15, 22, 31, 32], only one has conducted formal group comparisons [15]. This study reported better short-term pain and function outcomes for those with better pre-operative scores but contrary to the other evidence, also found the largest gains among this group of patients.

From the data available it is difficult for a surgeon to make an accurate prediction of how an individual patient is likely to recover post-operatively [33] and it is hard to know what to tell patients to expect [34]. It is unclear how pre-operative pain and function are likely to impact on the course of short- and long-term recovery and if any change beyond three months could be expected.

The aims of our study were to use information obtained from a cohort study to describe the trajectory of change after primary hip and knee arthroplasty, and to determine the effects of pre-operative pain and function on these trajectories and on outcomes one year after surgery.

## Methods

ADAPT is a single-centre UK prospective cohort study including people undergoing hip and knee arthroplasties. The study is registered on the NIHR Clinical Research Network Portfolio (UKCRN ID 8311). Its main purpose is to assess ways in which function can be assessed. The study was approved on 24^th^ December 2009 by the Southwest 4 Research Ethics Committee (09/H0102/72) and all patients provided informed, written consent.

Detailed information on study design, patient recruitment, inclusion-exclusion criteria, and assessment methods are provided in the published study protocol [35]. Briefly, between February 2010 and November 2011, patients waiting for hip or knee arthroplasty at a high-volume elective orthopaedic centre were invited to participate in the study. Patients were due to undergo a range of primary and revision arthroplasty procedures (primary total knee arthroplasty, revision total knee arthroplasty, unicompartmental knee arthroplasty, patellofemoral arthroplasty, primary total hip arthroplasty, revision total hip arthroplasty or hip resurfacing) so that functional measures could be investigated across a range of people with diverse indications for surgery and degrees of functional impairment. Exclusion criteria were assessed by a research nurse and included inability to provide written informed consent, to understand information about the study, to complete English language questionnaires (not all the questionnaires we used have been translated or validated for use in other languages), and severe functional limitations which would prevent completion of performance tests included in the protocol, but not reported here. In particular, patients unable to walk were excluded as this would have prevented the participant from attempting the functional tests.

Assessments were conducted prior to surgery (median number of days before surgery: hip 19 days; knee 11 days) and then at 3 and 12 months after surgery. At each post-operative assessment time, participants completed a postal questionnaire. Pain and functional ability were assessed by self-report using the Western Ontario and McMaster Universities Osteoarthritis index (WOMAC) function and pain sub-scales [36]. The WOMAC-function measure consists of 17 questions assessing the extent of function limitations when performing a range of daily activities. WOMAC-pain consists of five questions assessing pain during walking, using stairs, in bed, sitting or lying. Each sub-score ranges from 0–100 (worst to best). Data on gender, age, living arrangement, level of education, working status and number of joints affected by arthritis were collected in the pre-operative questionnaire. The type of surgical procedure undergone was extracted from participants’ medical records.

To ensure we had a sufficient number of patients to perform meaningful data analysis, we aimed to recruit approximately 250 patients for the main ADAPT study. However, the trajectory of recovery was expected to differ between patients listed for primary surgery and those listed for revision surgery. The latter participants were not considered in this research and analyses reported here focus on the group of ADAPT participants who underwent a primary arthroplasty. Analyses were conducted separately for patients undergoing hip and knee surgery. To investigate the influence of pre-operative function on the post-operative recovery pattern, patients were split into two groups: those with high or low pre-operative function using the median pre-operative WOMAC-function score as a cut-point. The median was chosen as a cut-point to prevent major imbalance by creating groups of equal size. A similar strategy was used for pain. Two participants undergoing hip surgery and one undergoing knee surgery who participated in the post-operative follow-up had missing pre-operative pain and function WOMAC scores. Their pre-operative high/low profiles were determined with a single imputation technique using regression models and the longitudinal measures of WOMAC-pain and function as independent factors.

We used univariate linear multi-level regression analysis, one for each outcome (WOMAC-pain and WOMAC-function) to model longitudinal trajectories (group changes) and conduct between-group comparisons of the change pattern. This approach accounts for repeated and unequal number of measurements per participant while producing estimations valid under the missing at random assumption [37]. Function and pain scores were standardised (using the pre-operative mean and standard deviation of the score of interest) to produce estimates which are comparable between regression models. The coefficients are interpreted per standard deviation change in the outcome of interest as the time changes by one month.

Change was modelled as two linear splines (a line between two points): one spline for the “short-term change” occurring between the pre-operative assessment and the second assessment (3 months post-operative) and another spline for the “long-term change” occurring between the two post-operative assessments (3 and 12 months). These changes were normally distributed (as revealed by residuals plots) allowing us to use the models presented above. The regression models were stratified by high/low pre-operative status. They were adjusted for the above two time splines, and random effects on the intercepts and slopes were modelled. Differences in the short-term or long-term changes between groups were tested using appropriate linear contrasts. Contrary to the changes between measurement points, outcome scores at each post-operative measurement occasion (3 months or 12 months) were non-normally distributed. Group comparisons of time-specific post-operative observed scores were therefore conducted with Mann-Whitney tests. A p-value of <0.05 was considered statistically significant. All models were fitted using Stata SE 13.1 and MLwiN v2.31 using Stata runmlwin command [38].

## Results

### Participants

Overall, 1451 eligible patients listed for hip or knee arthroplasty were approached about the ADAPT study and 264 agreed to take part (recruitment rates of 20% for patients waiting for hip surgery and 17% for those waiting for knee surgery). Of those who gave informed consent to participate, we excluded three people who did not subsequently undergo surgery and 12 people in whom all pre-operative data were unavailable. Patients who underwent a revision surgery (n = 85) were not considered for this study. Of those included in the final analysis, 80 had a primary total hip arthroplasty and 84 had knee arthroplasty (48 primary total knee arthroplasty and 36 unicompartmental knee arthroplasty).

At 12 months post-operative, 89% of the participants who underwent primary hip surgery and 86% of those who underwent primary knee surgery had complete WOMAC-pain and function scores. These rates were comparable by pre-operative high/low pain or function status using Fisher’s exact test: hip: pain p = 0.73, function p = 0.15; knee: pain p = 0.54, function p = 0.76. For hip surgery, three patients withdrew after their surgery, one after the three months assessment and five were lost to follow-up. For knee surgery, six patients withdrew after their surgery, two after the three months assessment and four were lost to follow-up.

The demographics and clinical characteristics of the cohort are shown in Table 1. The mean age was 65 years (SD = 11) for hip participants and 67 years (SD = 10) for knee participants. The median body mass index was 26 kg/m^2^ (25th = 24, 75th = 29) for hip participants and 31 kg/m^2^ (25th = 27, 75th = 35) for knee participants.

**Table 1:** Participant demographics and clinical characteristics.

		Hip (n = 80)		Knee (n = 84)	
		n	%	n	%
**Indication for surgery**	Osteoarthritis	74	92.5	81	96.4
	Other	6	7.5	3	3.6
**Surgery type**	Primary total joint arthroplasty	80	100.0	48	57.1
	Unicompartmental arthroplasty			36	42.9
**Gender**	Male	38	47.5	37	44.0
	Female	42	52.5	47	56.0
**Count other joints with osteoarthritis**	None	20	25.0	9	10.7
	One joint	22	27.4	22	26.2
	Two joints	13	16.3	13	15.5
	Three joints	8	10.0	13	15.5
	≥Four joints	13	16.3	23	27.4
	Unknown	4	5.0	4	4.7
**Living alone**	Living with someone	59	73.8	55	65.5
	Living alone	18	22.5	28	33.3
	Unknown	3	3.7	1	1.2
**Education**	Normal school leaving age	41	51.3	47	55.9
	College	20	25.0	24	28.6
	University	16	20.0	12	14.3
	Unknown	3	3.7	1	1.2
**Working status**	Full time	34	42.5	31	36.9
	Retired	38	47.5	46	54.8
	Unemployed	7	8.8	6	7.1
	Unknown	1	1.2	1	1.2

The distributions of participants by high or low pre-operative WOMAC-function and pain scores are presented in Table 2. For hip patients, the pre-operative function and pain median scores were 54 (25th = 38, 75th = 71) and 55 (25th = 30, 75th = 70) respectively; and for knee patients they were 51 (25th = 41, 75th = 66) and 45 (25th = 30, 75th = 60). Patients listed for a total knee arthroplasty and those listed for a unicompartmental arthroplasty had similar median scores at all assessment points for both pain (pre-operative p = 0.90; 3 months p = 0.11; 12 months p = 0.85) and function (pre-operative p = 0.77, 3 months p = 0.23; 12 months p = 0.66).

**Table 2:** Self-reported pain and function by site of surgery, assessment period and high/low function/pain profile.

		Hip (n = 80)					Knee (n = 84)				
		n	%	Median	25th, 75th^e^	p-value^f^	n	%	Median	25th, 75th	p-value^f^
**WOMAC function** ^**a**^^**,**^ ^**b**^	**Pre-operative-Total**	78	97.5	54	[38, 71]		83	98.8	51	[41, 66]	
	Missing	2					1				
	Low level of function	39	50.0	38	[29, 43]	<0.001	44	53.0	41	[35, 46]	<0.001
	Missing	1					0				
	High level of function	39	50.0	71	[62, 81]		39	47.0	66	[60, 75]	
	Missing	1					1				
	**3 months-Total**	76	95.0	90	[81, 96]		76	90.5	77	[65, 91]	
	Missing	4					8				
	Low level of function	37	48.7	87	[79, 93]	0.117	39	51.3	69	[53, 82]	<0.001
	Missing	3					5				
	High level of function	39	51.3	91	[84, 96]		37	48.7	88	[74, 96]	
	Missing	1					3				
	**12 months-Total**	71	88.8	96	[87, 100]		72	85.7	85	[65, 96]	
	Missing	9					12				
	Low level of function	33	46.5	94	[84, 99]	0.142	37	51.4	68	[54, 91]	<0.001
	Missing	7					7				
** **	High level of function	38	53.5	97	[94, 100]		35	48.6	94	[85, 97]	
	Missing	2					5				
**WOMAC Pain** ^**c**^^**,**^ ^**d**^	**Pre-operative-Total**	78	97.5	55	[30, 70]		83	98.8	45	[30, 60]	
	Missing	2					1				
	High level of pain	37	47.4	30	[25, 40]	<0.001	41	49.4	30	[20, 40]	<0.001
	Missing	0					0				
	Low level of pain	41	52.6	65	[60, 70]		42	50.6	60	[50, 70]	
	Missing	2					1				
	**3 months-Total**	76	95.0	95	[85, 100]		76	90.5	80	[58, 90]	
	Missing	4					8				
	High level of pain	34	44.7	95	[80, 100]	0.077	37	48.7	60	[45, 85]	0.001
	Missing	3					4				
	Low level of pain	42	55.3	100	[85, 100]		39	51.3	85	[75, 95]	
	Missing	1					4				
	**12 months-Total**	71	88.8	100	[90, 100]		72	85.7	85	[60, 100]	
	Missing	9					12				
	High level of pain	32	45.1	95	[85, 100]	0.126	34	47.2	68	[50, 90]	<0.001
	Missing	5					7				
	Low level of pain	39	54.9	100	[90, 100]		38	52.8	95	[80, 100]	
	Missing	4					5				

a. Function-Hip: High/low function status defined on pre-operative WOMAC-function using the median score (= 54) as cut-point.

b. Function-Knee: High/low function status defined on pre-operative WOMAC-function using the median score (= 51) as cut-point.

c. Pain-Hip: High/low pain status defined on pre-operative WOMAC-pain using the median score (= 55) as cut- point.

d. Pain-Knee: High/low pain status defined on pre-operative WOMAC-pain using the median score (= 45) as cut- point.

e. First and third quartiles: 25^th^ and 75^th^ percentiles

f. Mann-Whitney test to compare median scores by high/low function/pain profile.

### Hip arthroplasty

**Fig 1:**
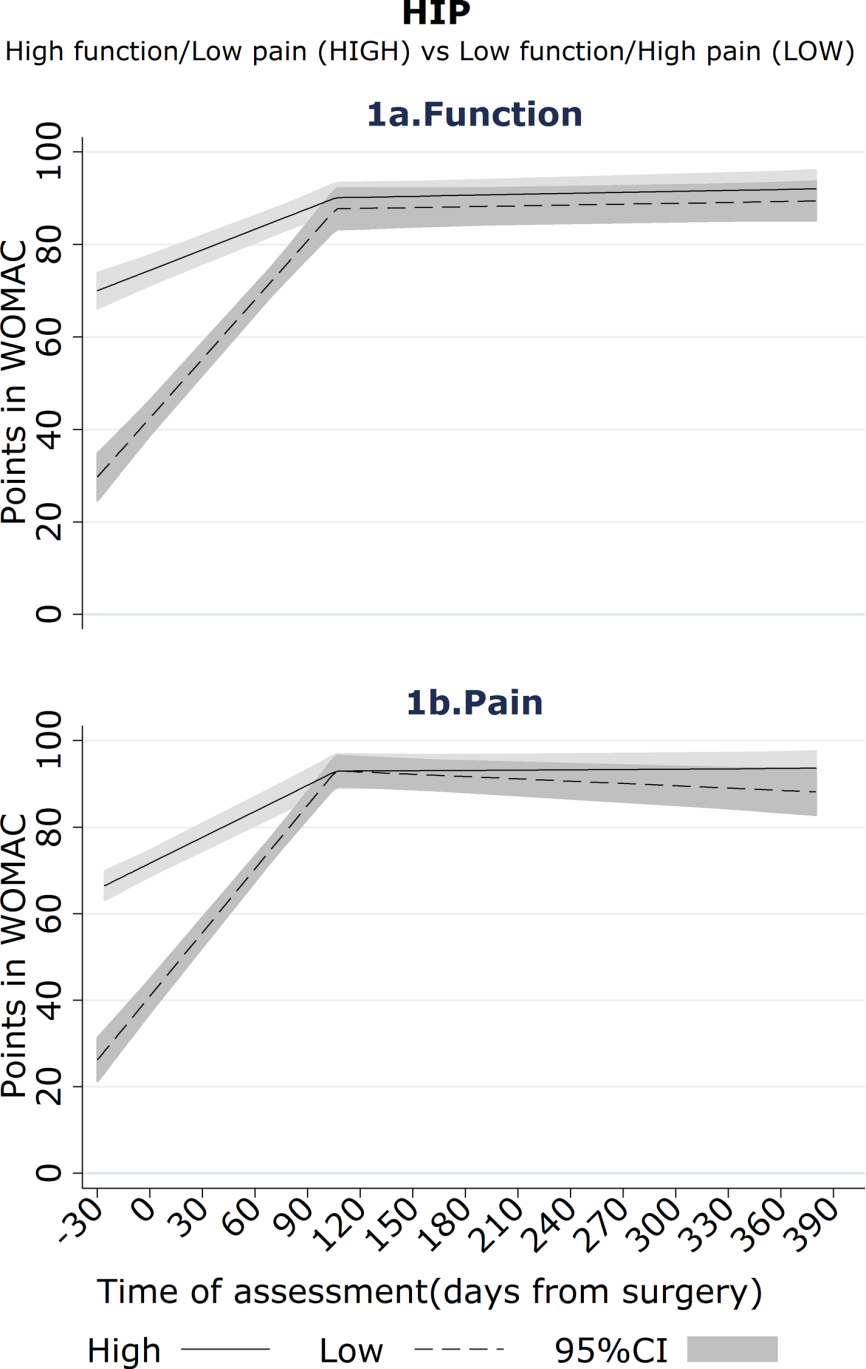
Hip-Mean trajectories^a^ for WOMAC-pain and WOMAC-function (Unstandardised outcomes) by high vs. low pre-operative pain/function^b^. A. The mean trajectories are derived from the fixed effects of linear mixed model stratified on high-low profile and regressing each outcome on the time of assessment parameterised as two linear splines. B. High/low function status defined on pre-operative WOMAC-function or -pain using their median scores (54 and 55) as cut-points.

Self-report functional ability and pain both improved after surgery, as shown in Fig 1 and Table 3.

**Table 3:** Hip^a^- Univariate linear mixed regression models of WOMAC-function or pain stratified by high/low pre-operative group profiles.

	Low group^b^				High group^c^				Low vs High^d^			
	Coef.	95% CI		p-value	Coef.	95% CI		p-value	Diff.	95% CI		p-value
**WOMAC-function**					** **			** **				
Pre-operative score^e^	**-0.491**	(-0.669,	-0.314)	<0.001	**1.003**	(0.851,	1.155)	<0.001	**-1.495**	(-1.728,	-1.261)	<0.001
Short-term change^f^	**0.596**	(0.516,	0.676)	<0.001	**0.207**	(0.160,	0.253)	<0.001	**0.390**	(0.297,	0.482)	<0.001
Long-term change^f^	0.000	(-0.000,	0.001)	0.514	0.000	(-0.000,	0.001)	0.263	0.000	(-0.001,	0.001)	0.885
**WOMAC-pain**					** **							
Pre-operative score^e^	**-0.471**	(-0.662,	-0.281)	<0.001	**1.013**	(0.867,	1.158)	<0.001	**-1.484**	(-1.724,	-1.244)	<0.001
Short-term change^f^	**0.712**	(0.643,	0.781)	<0.001	**0.291**	(0.242,	0.340)	<0.001	**0.421**	(0.336,	0.506)	<0.001
Long-term change^f^	-0.001	(-0.002,	0.000)	0.052	0.000	(-0.001,	0.001)	0.722	-0.001	(-0.002,	0.000)	0.078

Coefficient with p-value<0.05 are highlighted in bold.WOMAC- function and–pain are modelled as standardised outcomes.

a. The regression coefficients are derived from random intercept and slope models adjusted for time of assessment parameterised as two linear splines (short-term changes and long-term changes). The variances of random effects and correlation coefficients are not presented but are available on request. High/low function status defined on pre-operative WOMAC-function or -pain using their median scores (54 and 55) as cut-points.

b. The low group refers to participants with low functional ability or high level of pain.

c. The high group refers to participants with high level of function or low level of pain before surgery.

d. Difference between the low and high groups’ regression coefficients assessed with linear contrasts.

e. Intercept: Estimated mean function or pain standardised score on the day of surgery.

f. Short-term and long-term changes: Estimated monthly mean change in function or pain standardised scores between the pre-operative and first post-operative assessments (~3 months) or between the first and second post-operative assessments (~3 and ~12 months), respectively.

Improvements in function and pain mainly occurred within the first 3 months. Patients with low pre-operative function experienced short-term functional improvements that were larger than those reported by patients with high pre-operative function, between-group difference in monthly mean change in WOMAC-function standardised score 0.39 (95% CI 0.30, 0.48; p<0.001, Table 3). No evidence of further improvement in function between 3 months and 12 months post-operative was found for either group. The absolute level of function at 3 months was comparable between the high function group and the low function group (p = 0.12, Table 2). At 12 months, patients with low self-reported pre-operative function had reached a level of function similar to that reported by the patients with higher pre-operative function, observed median WOMAC-function scores in the low pre-operative function group 94 compared with 97 in the high pre-operative function group (p = 0.14), Table 2.

Short-term pain improvements were also larger for those with high pre-operative pain, between-group difference in monthly mean change in WOMAC-pain standardised score 0.42 (95%CI 0.34, 0.51, Table 3; p<0.001). No evidence of further improvement in pain between the 3 and 12 month post-operative scores was found for either group. Absolute levels of WOMAC-pain were similar between groups at 3 months (p = 0.08, Table 2) and 12 months (p = 0.13, Table 2) post-operative.

### Knee arthroplasty

**Fig 2:**
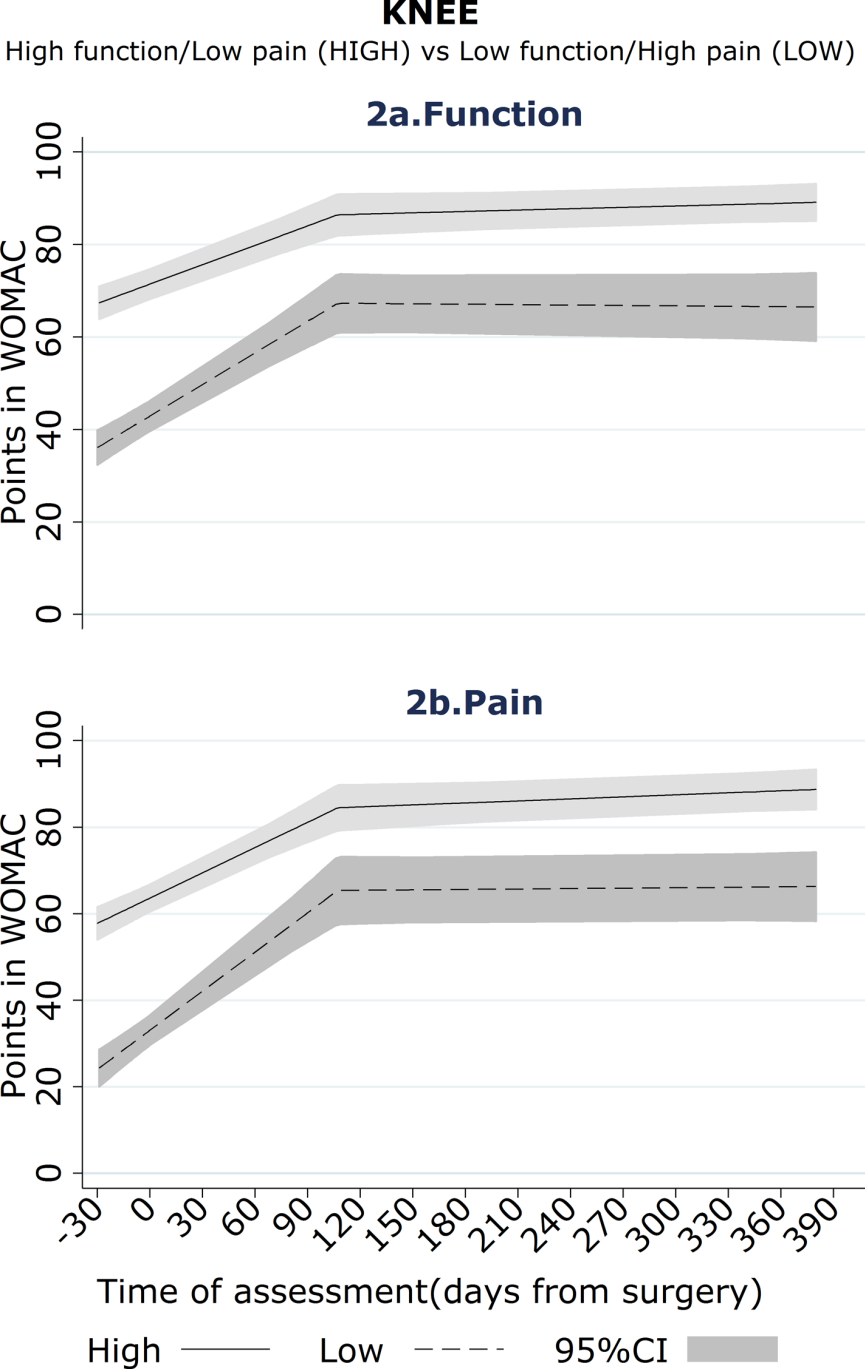
Knee -Mean trajectories^a^ for WOMAC-pain and WOMAC-function (Unstandardized outcomes) by high vs. low pre-operative pain/function^b^. A. The mean trajectories are derived from the fixed effects of linear mixed model stratified on high-low profile and regressing each outcome on the time of assessment parameterised as two linear splines. B. High/low function status defined on pre-operative WOMAC-function or -pain using their median scores (51 and 45) as cut-points.

Patients experienced an improvement in self-reported function and pain after their knee surgery, with this improvement occurring primarily in the first three months, as shown in Fig 2 and Table 4.

**Table 4:** Knee^a^- Univariate linear mixed regression models of WOMAC-function or–pain stratified by high/low pre-operative group profiles.

	Low group^b^				High group^c^				Low vs High^d^			
	Coef.	95% CI		p-value	Coef.	95% CI		p-value	Diff.	95% CI		p-value
**WOMAC-function**					** **			** **				
Pre-operative score^e^	**-0.555**	(-0.733,	-0.378)	<0.001	**0.995**	(0.819,	1.172)	<0.001	**-1.551**	(-1.802,	-1.300)	<0.001
Short-term change^f^	**0.373**	(0.286,	0.459)	<0.001	**0.229**	(0.173,	0.285)	<0.001	**0.144**	(0.041,	0.247)	0.006
Long-term change^f^	0.000	(-0.001,	0.001)	0.827	0.001	(-0.000,	0.001)	0.179	-0.001	(-0.002,	0.001)	0.350
**WOMAC-pain**					** **							
Pre-operative score^e^	**-0.595**	(-0.770,	-0.420)	<0.001	**0.962**	(0.802,	1.122)	<0.001	**-1.557**	(-1.794,	-1.319)	<0.001
Short-term change^f^	**0.464**	(0.355,	0.572)	<0.001	**0.299**	(0.225,	0.372)	<0.001	**0.165**	(0.034,	0.296)	0.014
Long-term change^f^	0.000	(-0.001,	0.001)	0.737	0.001	(-0.000,	0.002)	0.074	-0.001	(-0.002,	0.001)	0.388

Coefficient with p-value<0.05 are highlighted in bold. WOMAC- function and–pain are modelled as standardized outcomes.

a. The regression coefficients are derived from random intercept and slope models adjusted for time of assessment parameterised as two linear splines (short-term changes and long-term changes). The variances of random effects and correlation coefficients are not presented but are available on request. High/low function status defined on pre-operative WOMAC-function or -pain using their median scores (51 and 45) as cut-points.

b. The low group refers to participants with low functional ability or high level of pain.

c. The high group refers to participants with high level of function or low level of pain before surgery.

d. Difference between the low and high groups’ regression coefficients assessed with linear contrasts.

e. Intercept: Estimated mean function or pain standardised score on the day of surgery.

f. Short-term and long-term changes: Estimated monthly mean change in function or pain standardised scores between the pre-operative and first post-operative assessments (~3 months) or between the first and second post-operative assessments (~3 and ~12 months), respectively.

Patients with low pre-operative function reported short-term functional improvements that were larger than those reported by patients with high pre-operative function, between-group difference in monthly mean change in WOMAC-function standardised score 0.14 (95%CI 0.04, 0.25; p <0.01, Table 4. No evidence of further improvement in function between 3 months and 12 months post-operative was found for either group. In contrast to the results for hip patients, knee patients with low pre-operative scores reported significantly worse scores for function at 3 months (p <0.001, Table 2) and 12 months, observed WOMAC-function median score 68(25th = 54, 75th = 94) for patients with low pre-operative function compared with 94 (25th = 85, 75th = 97) for patients with high pre-operative function (p <0.001, Table 2).

Short-term pain improvements in pain were also larger for participants with high pre-operative pain compared to those with low pre-operative pain, between-group difference in monthly mean change in WOMAC-pain standardised score 0.17 (95%CI 0.03, 0.30; p = 0.014, Table 4). However, the pain levels reached at 3 months (p = 0.001, Table 2) and 12 months post-operative were dependent on the pre-operative pain status with higher post-operative pain for participants with high pre-operative pain, observed WOMAC-pain median score 68(25th = 50, 75th = 90) for patients with high pre-operative pain compared with 95(25th = 80, 75th = 100) for patients with low pre-operative pain (p<0.001), Table 2).

## Discussion

The majority of the symptomatic gain made after both hip and knee arthroplasty was achieved within the first three months after surgery. No significant mean change beyond three months was observed in our sample. Patients with worse pain and functional limitations prior to surgery had the greatest capacity to benefit from arthroplasty. They had larger improvements during the first three months of their surgery than those with better pre-operative scores; beyond this period, the mean changes in pain and function were not related to pre-operative scores. One year after surgery, the levels of pain and function achieved by those who underwent a primary total hip arthroplasty were not dependent on pre-operative pain and function. In contrast, patients who had the most severe pain and functional impairment prior to knee arthroplasty never achieved as good an outcome as those with less severe pre-operative pain and functional limitations.

Our results on improvements in function and pain after surgery are in agreement with previous studies which show that most of the recovery as measured by self-reported outcomes occurs in the first few months after arthroplasty [6, 8–13, 16, 39]. Our findings based on robust statistical modelling and tests, in conjunction with existing evidence, suggest that no or only small clinical gains can be expected beyond three months for the majority of patients. This might define a critical phase in the recovery process at the end of which the course of improvement should be assessed to identify patients with no or very little change requiring more intensive clinical care. This is also an important period to consider when exploring post-operative recovery by level of pre-operative symptoms. The patterns of recovery of those with high and low pre-operative scores only differed during the first three months following the surgery and the long-term recovery has very little impact on the difference/absence of difference between those two groups observed at 12 months.

We have found that the associations between pre-operative pain and function scores and the course of recovery differ between patients undergoing hip and knee arthroplasty. Our findings showing that the level of pre-operative pain/function influences the course of recovery and level of score reached at 12 months after knee surgery is consistent with previous research [11, 15, 17, 28, 40]. Knowing the optimal time to undertake surgery is important, as potential benefits have to be weighed against the risks of surgery, and not everyone will benefit. Previous studies have shown that between 7 and 23% of patients have an unfavourable long-term pain outcome after hip arthroplasty and 10 to 34% after knee arthroplasty [4]. Our study suggests that for patients with knee arthroplasty it might be better to do the surgery when symptoms are less severe, i.e. earlier rather than later in the course of the disease, although it is not clear exactly what the ‘cut-off’ point, in terms of symptomatic severity, should be. This also suggests the potential value of more intensive and comprehensive pre- and post-operative rehabilitation for patients with the most severe symptoms prior to their knee surgery to achieve better post-operative outcomes. The pre- and post-operative treatment received by participants was not documented in this study. All patients were offered standard care as provided at the treating centre. This comprised a pre-operative educational class focusing on preparation for surgery and the hospital stay, and post-operative outpatient physiotherapy on a needs basis.

The absence of an association between the pre-operative score and post-operative pain and function outcomes following hip arthroplasty is in disagreement with several studies [15, 22, 28, 40] or in partial agreement with other study with similar findings for pain but not function outcome [32]. Disagreement could be explained by the timing of the final outcome assessment, 4, 6 or 24 months after surgery [15, 28, 32] vs. 12 months in our study, but not always [40] and by the nature of the patient reported outcome measure considered [22]. Our study is, however, not the first to have found no evidence of an association between pre-operative scores and post-operative outcomes following hip arthroplasty [27, 29].

The absence of significant improvements observed after three months may be a consequence, at least in part, from the ceiling effect inherent in patient reported outcome measures used in arthroplasty research [41–46]. Patient reported outcome measures are defined within a set range of possible scores, which limits the ability of the questionnaire to detect improvement beyond the bounds of the questionnaire[47, 48] and gives more room for improvement in patients who start at the bottom of the score than for those who are closer to its upper limit, favouring the observation of a greater recovery for patients with severe pre-operative symptoms (i.e. low scores). The absence of a statistically significant difference between the high and low pain or function groups 12 months after hip arthroplasty could reflect this ceiling effect of the WOMAC scores, forcing the two groups towards a common destination rather than an actual absence of difference. On the contrary, the differences observed between the high and low groups 12 months after knee surgery are likely to be conservative and might have been larger in the absence of ceiling effect.

The strengths of the study include the use of robust and validated outcome measures, good follow-up rates, more than one follow-up point, and a reasonable sample size for this type of investigation. The use of linear mixed regression models allowed the modelling of the repeated measures of pain and function and facilitated the use of all available observations including those of patients who did not participate in all follow-ups while providing estimations valid under the missing at random assumption.

The apparent limitation of a relatively low participation rate is not, in our view, a major problem because this was not a trial, the overall demographics of those taking part are similar to those found in other studies, and we achieved the wide variation in disease severity at baseline which we were aiming for. Participation rates are explained by the high burden of attending additional research appointments in the ADAPT study. It is possible that those who agreed to take part differed from those who declined with regard to important determinants of the outcome of arthroplasty, such as mental health. Furthermore, this cohort is a single centre study limiting its external validity as context and culture may influence outcomes [49].

The modest sample size limited our ability to adjust for factors known to be associated with post-operative outcomes such as age, gender, mental health and co-morbidities [39, 50, 51]. However, the lack of adjustment does not prevent using our findings to investigate descriptive research aims like ours describing and plotting the pattern of recovery by high and low status. A larger sample would nevertheless be required to adjust for confounders and investigate aetiological research questions.

We analysed results of patients who underwent unicompartmental and total knee arthroplasty together to keep a large enough study group. Evidence suggests that following unicompartmental knee surgery there is a faster recovery and lower rates of adverse events [52, 53]. Others have found comparable post-operative pain and function outcomes [54]. In our sample, those two groups of patients had similar pre- and post-operative WOMAC median scores at all assessment points (median pre-operative pain: unicompartmental 45 vs. total replacement 40; at 3 months post-operative: 83 vs 76; at 12 months post-operative: 85 vs 88; respectively 51 vs 51, 80 vs 73 and 86 vs 85 for function; all p-values>0.05), suggesting similar patterns of change and the acceptability of grouping them together. Moreover, patients who underwent unicompartmental surgery were equally split between the high and low pain/function groups; their impact on the pattern of change was therefore similar in each group.

Measuring outcomes only at 3 and 12 months is another limitation of the study, as inclusion of additional assessment points would have allowed more detailed investigation of recovery trajectories. We considered that additional assessment points would be too much of a burden for participants with a probability of increased levels of attrition.

Losina and Katz [55] discussed the difference between the journey (the gains made) and the destination (the final outcome) after arthroplasty, suggesting that those with severe pre-operative symptoms improve the most (have the best journey), but can have the worst final outcome (worst destination). It appears from our findings that for self-reported pain and function this does not hold true for hip arthroplasty, but does for knee arthroplasty.

## Conclusions

Most of the improvement following hip and knee arthroplasty occurs within the first three post-operative months with no subsequent statistically significant improvement. Patients with worse pre-operative function or pain report poorer outcome at 12 months after knee arthroplasty, but not hip arthroplasty. Further investigations are now required to determine if patients with severe symptoms at the time of their knee arthroplasty have a different pre-surgical history than those with less severe symptoms and if they could beneficiate from earlier surgical intervention, when symptoms are less severe, and/or tailored pre- and post-operative rehabilitation to achieve better post-operative patient-reported outcomes.
